# A Rare Case of a Panic Attack Inducing a Junctional Escape Rhythm on ECG

**DOI:** 10.7759/cureus.40677

**Published:** 2023-06-20

**Authors:** Adam Kurnick, Alam Ahmed, Joseph Gonzalez, Adam S Budzikowski

**Affiliations:** 1 Department of Internal Medicine, The State University of New York (SUNY) Downstate Health Sciences University, Brooklyn, USA; 2 Division of Cardiovascular Medicine - Electrophysiology (EP) Section, The State University of New York (SUNY) Downstate Health Sciences University, Brooklyn, USA

**Keywords:** electrocardiogram (ecg/ekg), cardiac stress test, panic disorder, panic attack, junctional escape rhythm

## Abstract

We present a unique and rare case of a young female patient who presented with complaints typical of her prior panic attacks and was found to have a junctional escape rhythm on ECG. Upon resolution of her symptoms, a repeat ECG demonstrated a return to normal sinus rhythm. Given that alternative etiologies had been ruled out, it was postulated that her panic attack induced a transient junctional escape rhythm.

## Introduction

A junctional rhythm typically occurs in the setting of a transient dysfunction of the sinoatrial (SA) node and can be caused by various conditions or medications. Associated conditions include electrolyte disturbances, thyroid disorders, cardiac surgery, medications, and more [1]. There have been prior reports of junctional rhythms associated with supraventricular tachyarrhythmias [2-6]; however, upon literature review, there appears to be no previously documented association with panic disorder. A panic attack can manifest in symptoms that appear to be related to the heart, such as palpitations, shortness of breath, or chest pain.

We present the unique case of a young woman who presented with a junctional escape rhythm precipitated by a panic attack. In this case, inherent sinus node dysfunction as well as other potentially responsible etiologies were ruled out.

## Case presentation

A 21-year-old woman with a past medical history of anxiety and panic attacks presented to the emergency department with complaints of chest tightness/ pain, shortness of breath, and perioral and fingertip tingling, all symptoms consistent with her previous panic attacks. She had no prior cardiac history and no family history of cardiac disease. She worked as a cashier, smoked hookah occasionally, drank alcohol socially (none prior to admission), and denied using other substances. Upon presentation to the emergency department, she was afebrile and normotensive; her heart rate was 63 bpm, and she was mildly tachypneic at 20 breaths per minute. Her physical examination revealed a young woman in no distress with a regular rate and rhythm, normal heart sounds, and no murmurs. The rest of her examination was unrevealing. Laboratory tests (Table 1) were unremarkable, other than an elevated total bilirubin of 1.6 mg/dL and a mild increase in serum creatinine. A urine drug screen was negative, and human chorionic gonadotropin (hCG) was undetectable. Thyroid function tests were normal, and troponins were within normal limits. The COVID screen was negative.

**Table 1:** Laboratory blood and urine test results

Test name		Value	Reference range	Units
Serum	White blood cells	4.80	4.50 – 10.90	K/uL
	Hemoglobin	15.0	12.0 – 16.0	g/dL
	Platelets	261	130 – 400	K/uL
	Sodium	139	136 – 146	mmol/L
	Potassium	4.3	3.5 – 5.0	mmol/L
	Magnesium	2.10	1.60 – 2.60	mg/dL
	Phosphorus	3.4	2.5 – 4.5	mg/dL
	Creatinine	1.05 (high)	0.50 – 0.90	mg/dL
	Aspartate aminotransferase	16	10 – 35	U/L
	Alanine transaminase	11	0 – 31	U/L
	Total bilirubin	1.6 (high)	0.0 – 1.2	mg/dL
	Troponin	0.010	≤ 0.010	ng/mL
	Thyroid-stimulating hormone	1.510	0.270 – 4.200	uIU/mL
	Free thyroxine (T4)	1.65	0.93 – 1.70	ng/dL
	Human chorionic gonadotropin (hCG)	< 0.5	0.0 – 5.0	mIU/mL
Urine	Drug screen	Not detected	Barbituates; benzodiazepines; bocaine; methadone; opiates	n/a

**Figure 1:**
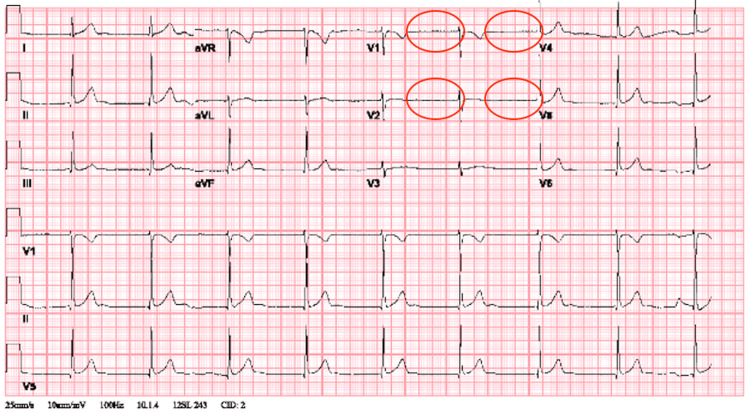
ECG on presentation, demonstrating a junctional escape rhythm at 53 bpm with the absence of p waves (circles) ECG: electrocardiogram; bpm: beats per minute

**Figure 2:**
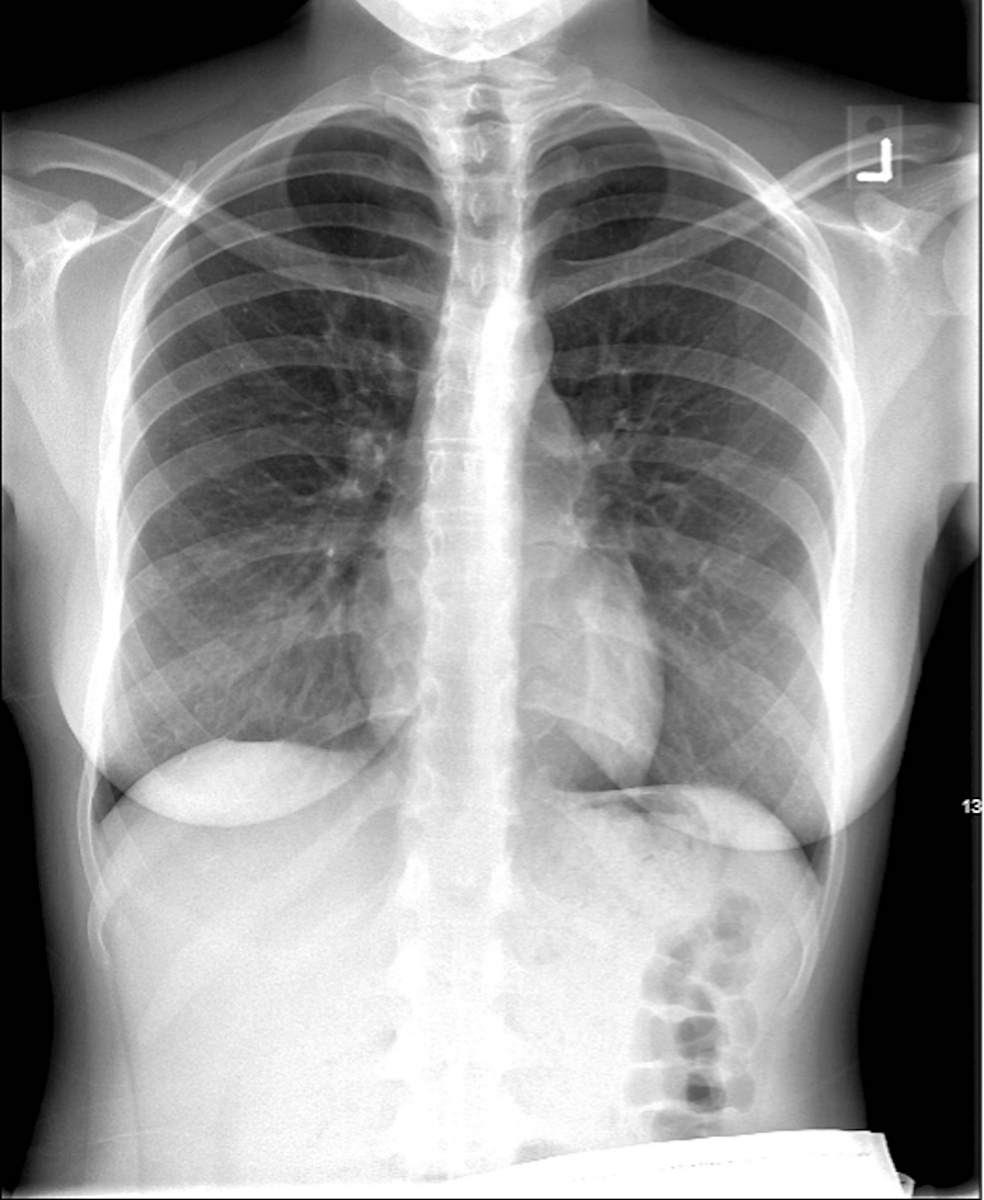
Posteroanterior (PA) chest x-ray reveals no abnormalities

The initial electrocardiogram (ECG) revealed a junctional escape rhythm at 53 bpm (Figure 1), and a chest x-ray revealed no abnormalities (Figure 2).

**Figure 3:**
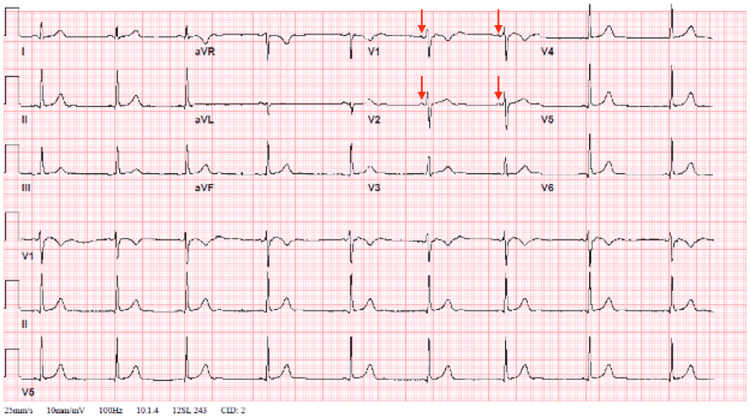
ECG upon resolution of symptoms demonstrates sinus rhythm (at 53 bpm), replacing junctional rhythm. Arrows are pointing to p waves not present on the initial ECG. ECG, electrocardiogram; bpm: beats per minute

While in the emergency department, the patient's symptoms self-resolved. A repeat ECG was then performed, which revealed the resolution of the junctional escape rhythm and instead demonstrated sinus bradycardia with a heart rate of 53 bpm (Figure 3).

An exercise stress test was performed as well, which showed a normal chronotropic response and was negative for stress-induced chest pain or angina, ischemic changes, exercise-induced arrhythmias, or arrhythmias during recovery.

## Discussion

To our knowledge, this is the first reported case of junctional rhythm caused by a panic attack. Junctional rhythm typically results from a transient dysfunction of the sinoatrial (SA) node and has a host of etiologies ranging from medication-induced to inherited disorders, trauma, and many more [1]. Upon a literature review, we found that the incidence and prevalence of junctional rhythms in the general population are not well defined. Junctional rhythm is characterized by the presence of regular, narrow QRS complexes (or with a preexisting bundle branch block) without visible P waves or retrogradely conducted P waves (negative in limb leads) either preceding, coinciding, or following the QRS complexes. Patients may present with dizziness, fatigue, presyncope, syncope, or neck pulsation because the contraction of the atria is occurring simultaneously with that of the ventricles. Some patients may be asymptomatic. Based on the ECG findings, it is convincing that our patient presented with a junctional escape rhythm at a rate of 53 bpm. Other causes potentially responsible for junctional rhythm were excluded, including recent cardiac surgery, hypothyroidism, electrolyte abnormalities, and potentially offending medications. The troponin was negative, and the ECG did not reveal ischemic changes, ruling out acute coronary syndrome as a potential etiology. Furthermore, the exercise stress test revealed excellent chronotropic response, as the patient was able to reach peak heart rate (HR) at 91% of her age-predicted max (181 bpm), pointing to the transient nature of sinus node dysfunction. Given that our workup did not reveal a potential cause for the junctional rhythm and the transient ECG findings correlated with the patient's symptoms of a panic attack, we believe that the junctional rhythm was caused by the patient's panic attack.

Upon literature review, we discovered prior reports of panic disorders or episodes of emotional stress-inducing events being associated with paroxysmal supraventricular tachyarrhythmias but not with junctional rhythm [2-6]. Supraventricular tachyarrhythmias generally present with a rapid heart rate of more than 100 beats per minute, whereas a junctional rhythm typically has a slower heart rate of 40-60 beats per minute, as in our patient. It is not clear how panic disorders can cause transient suppression of the SA node and the emergence of junctional rhythm; one can postulate that dysregulation of stress hormones and neurotransmitters may be a contributing factor. Other conditions that may precipitate vagal reflexes, namely right ventricular strain and activation of the Bezold-Jarisch reflex, may have a similar presentation, although we do not believe those to be present here.

## Conclusions

We conclude that the junctional rhythm seen on presentation is an unusual consequence of a panic attack. Although panic attacks have been linked to supraventricular tachyarrhythmias, to our knowledge, this is the first case of a junctional rhythm caused by a panic attack. Further studies are warranted in order to explore this phenomenon.
